# Simplification of ankle-brachial-index measurement using Doppler-waveform classification in symptomatic patients suspected of lower extremity artery disease

**DOI:** 10.3389/fcvm.2022.941600

**Published:** 2022-09-09

**Authors:** Antoine Métairie, Quentin Tollenaere, Damien Lanéelle, Alexis Le Faucheur, Estelle Le Pabic, Loukman Omarjee, Guillaume Mahé

**Affiliations:** ^1^Vascular Medicine Unit, CHU Rennes, Rennes, France; ^2^Vascular Medicine Unit, CHU Caen-Normandie, Caen, France; ^3^University of Rennes, Rennes, France; ^4^CHU Rennes, Inserm, Clinical Investigation Center (CIC), Rennes, France; ^5^Pôle Imagerie Médicale et Explorations Fonctionnelles, Hôpital Pontchaillou, Rennes, France

**Keywords:** lower extremity artery disease, Doppler waveforms, ankle-brachial index, peripheral arterial disease, claudicant

## Abstract

**Objectives:**

Ankle-brachial index (ABI) is commonly used for screening lower extremity peripheral artery disease (PAD) according to the international guidelines. Arterial Doppler waveform recordings is a tool to diagnose and assess PAD severity. We hypothesized that ABI measurement could be simplified by measuring only the pressure where the best arterial flow is recorded. The aim of this study was to evaluate the concordance between ABI performed according to the American Heart Association guidelines (AHA-ABI) and ABI measured according to best arterial waveform (FLOW-ABI).

**Design:**

This was a monocentric cross-sectional study.

**Methods:**

We included patients with exertional limb symptoms suspected of PAD. Arterial Doppler waveforms and ABI were acquired on both lower extremities at the pedis and tibial posterior arteries. Each arterial waveform was classified using the Saint-Bonnet classification. Concordances were analyzed with the kappa coefficient (confidence interval 95%). Exercise PAD study was registered n° NCT03186391.

**Results:**

In total, one hundred and eighty-eight patients (62+/−12 years and 26.8+/−4.5 kg/m^2^) with exertional limb symptoms were included from May 2016 to June 2019. On each extremity, FLOW-ABI had excellent concordance for the diagnosis of PAD with the AHA-ABI with a kappa of 0.95 (95% CI: 0.90, 0.99) in the right extremity and 0.91 (95% CI: 0.86, 0.97) in the left extremity.

**Conclusion:**

There is almost perfect concordance between AHA-ABI and FLOW-ABI. Thus, ABI can be simplified into five pressure measurements instead of seven in patient suspected of PAD with exertional limb symptoms. The question remains in patients with chronic limb ischemia.

## Introduction

Lower extremity peripheral artery disease (PAD) is one of the most common diseases with a major cardiovascular morbi-mortality, particularly in developed countries (1). Its prevalence is estimated around 236 million persons worldwide in 2015 (1, 2), with a growth trend. The estimated prevalence in Europe and the United States of America is between 3 and 10% according to the different studies and increases with age (between 15 and 20% over 70 years old) (3).

One of the recommended diagnostic means is the measurement of the ankle-brachial index (ABI) at rest by dividing the highest of the two arterial pressures of the two ankle arteries by the highest brachial pressure between both arms (4–8). To be considered as pathological, a cutoff equal to 0.90 and below was retained (4, 5). In addition to its diagnostic utility, ABI has a prognostic interest with, when pathological, more than doubling of the 10-year rates of coronary events, cardiovascular mortality, and total mortality (9, 10). Guidelines about ABI were proposed in 2012 by the *American Heart Association* to standardize the procedure (11). The ABI is based on counterclockwise sequence of seven measures of pressure. This specific sequence to measure ABI is not done in clinical practice due to several barriers including the measurement duration, lack of reimbursement, and staff availability (12). Indeed, the average time for an ABI measurement is around 5 min for a trained physician and without considering the resting time before the measurement (12) whereas the primary care physician consultation time is limited (16 min in 2006 in France to <10 min in numerous countries) (13–16). A simplification (i.e., shorter duration) in the measurement could be interesting for the diffusion and the use of this diagnostic method.

The arterial flow analysis of the Doppler waveform is as well an interesting tool for the arterial hemodynamic evaluation (7, 17, 18), and can be an interesting diagnostic tool in particular populations such as patients with chronic kidney disease and diabetes (19) where the ABI value can be falsely elevated due to arterial calcifications (20).

We hypothesized that the highest arterial pressure in each extremity is located on the distal artery with the best arterial Doppler waveform; thus, ABI measurement might be simplified by measuring only five pressures instead of the seven as proposed by the AHA and other international guidelines (6, 7, 11, 21).

The main objective of this study was to analyze the concordance for the diagnosis of PAD between the ABI measured with the AHA method (AHA-ABI) and ABI measured considering the pressure in the artery with the best Doppler waveform (FLOW-ABI) to simplify the measurement in clinical practice.

## Materials and methods

### Study design and population

This is a retrospective study on consecutive patients suspected of PAD with exertional limb pain referred between May 2016 and June 2019 to our vascular center in the University Hospital of Rennes, France (22).

The study was approved by an institutional review board from the University Hospital of Rennes (12, 17). All participants gave written informed consent. The study protocol conforms to the ethical guidelines of the 1975 Declaration of Helsinki. The exercise PAD study was registered with the American National Institutes of Health database under reference n° NCT03186391.

Patients were included if they had full data available including arterial Doppler waveform and ABI measurements available for both extremities.

### Demographic characteristics

We collected all the medical history including age, sex, body mass index, comorbidities, and medications (statins, antihypertension treatment, anticoagulant, or antiplatelet).

### Doppler waveform analysis and ABI measurement

Patients were at rest in a comfortable temperature room (20–22°C) for at least 20 min, in a supine position prior to testing (23). Arterial Doppler waveforms were described for each artery of the limbs before pressure measurements with a hand-held Doppler probe (8 MHz; Basic Atys Medical, Soucieu en Jarrest, France). Arterial Doppler waveforms were described using the Saint-Bonnet Classification as recommended by the French Vascular Teachers of Vascular Medicine (7, 17, 18). This classification provides a superior categorization rate when compared to other classifications (24). A normal flow consists of a multiphasic curve (N or A among Saint-Bonnet) (8, 17). In the case of an arterial lesion, the arterial waveform is modified depending on the degree of arterial lumen stenosis allowing the assessment of the severity of the disease and the state of collateral arteries. Saint-Bonnet classification ranges from type N to E until 0 where N stands for normal, type E describes the type of waveforms recorded in highly pathological arteries, and 0 describes an occluded artery. In the simplified classification, there are six types: Saint-Bonnet N to Saint-Bonnet A (considered as a normal flow), Saint-Bonnet B, Saint-Bonnet CD, Saint-Bonnet E, and Saint-Bonnet 0 (no flow). The classification was developed to homogenize the Doppler arterial flow description (17). Table 1 depicts the classification and its equivalence to the classification proposed by the Society for Vascular Medicine (SVM) and Society for Vascular Ultrasound (SVU) consensus (25, 26).

**Table 1:** Equivalence between Saint-Bonnet classification and SVM/SVU classification.

**Saint-Bonnet classification**	**Doppler waveforms**	**SVM/SVU classification**
**Arterial waveform with high resistive flow**		
Saint-Bonnet N	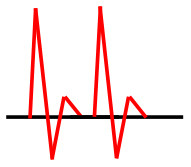	Multiphasic (Triphasic), high resistive with sharp peak, and rapid upstroke
Saint-Bonnet A	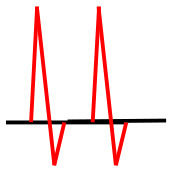	Multiphasic (Biphasic), high resistive with sharp peak, and rapid upstroke
Saint-Bonnet B	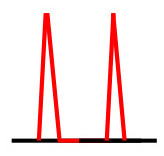	Monophasic, high resistive with sharp peak, and rapid upstroke
Saint-Bonnet CD	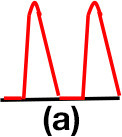 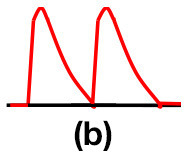 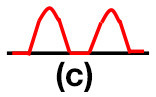	Monophasic, high resistive, (a) Dampened (b) Dampened, and prolonged upstroke (c) Dampened, and prolonged upstroke
Saint-Bonnet E	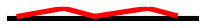	Monophasic, high resistive, dampened, and prolonged upstroke
Saint-Bonnet 0	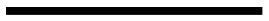	Absent
**Arterial waveforms with intermediate or low resistive flow**		
Saint-Bonnet N-CF	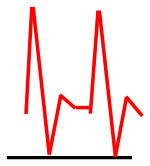	Monophasic, intermediate resistive with sharp peak, and rapid upstroke
Saint-Bonnet A-CF	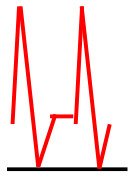	Monophasic, intermediate resistive with sharp peak, and rapid upstroke
Saint-Bonnet B-CF	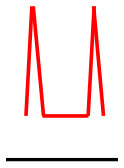	Monophasic, low resistive with sharp peak, and rapid upstroke.
Saint-Bonnet CD-CF	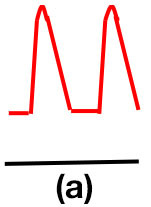 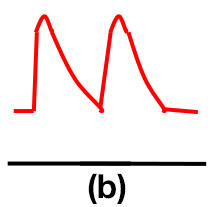 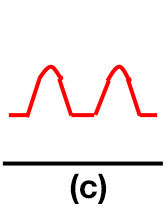	Monophasic, low resistive, (a) Dampened (b) Dampened, and prolonged upstroke (c) Dampened, and prolonged upstroke
Saint-Bonnet E-CF	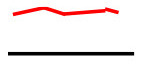	Monophasic, low resistive, dampened, and prolonged upstroke

Please refer to validation papers for both classifications (17, 25). SVM means Society for Vascular Medicine. SVU means Society for Vascular Ultrasound.

The AHA-ABI was calculated as proposed by AHA guidelines: “During the sequence of measurement, the first measurement should be repeated at the end of the sequence and both results averaged to temper the white coat effect of the first measurement, except if the difference between the 2 measurements of the first arm exceeds 10 mmHg. In that case, the first measurement should be disregarded and only the second measurement should be considered. For example, when the counterclockwise sequence — right arm, right posterior tibial artery (RPTA), right dorsalis pedis artery (RDPA), left posterior tibial artery (LPTA), left dorsalis pedis artery (LDPA), left brachial artery — is used, the measurement of the right arm should be repeated at the end of the sequence and both results obtained at the right arm should be averaged unless the difference between the 2 measurements of the right arm exceeds 10 mm Hg. In this case, only the second measurement of right arm pressure should be considered” (11, 12).

The FLOW-ABI was obtained by dividing the pressure of the lower extremity artery with the best Doppler waveforms by the highest brachial pressure between both arms. In the case of similar Doppler waveform between the posterior tibial artery (PTA) and the dorsalis pedis artery (DPA), we used the posterior tibial artery by default.

The last three types (CD, E, and 0) were grouped together due to their small numbers to have a significantly large group for comparison.

### Statistical analyses

The results are expressed as mean ± standard deviation in the case of normal distribution (Shapiro–Wilk test) or in median [25th centile and 75th centile] in the other cases. The Kruskal– Wallis test (KW) was used for the comparisons between the ankle blood pressures and the Saint-Bonnet Doppler waveform types, analyzing independently each limb. The concordance between the AHA-ABI and the FLOW-ABI for the diagnosis of PAD was assessed using the Kappa coefficient expressed with a confidence interval of 95% for the right and left limbs. A second analysis was performed in a subgroup of limbs that had different Doppler waveform types. The Landis and Koch interpretation of kappa values was used: 0.21–0.40: fair; 0.41–0.60: moderate, 0.61–0.80: substantial; >0.80: almost perfect (27). Correlations between ABI-AHA and FLOW-ABI were assessed with the Spearman's correlation coefficient. The significance level used for all statistical tests was <0.05. All analyses were performed with SAS software, v.9.4^®^ (SAS Institute, Cary, NC, USA).

## Results

**Figure 1:**
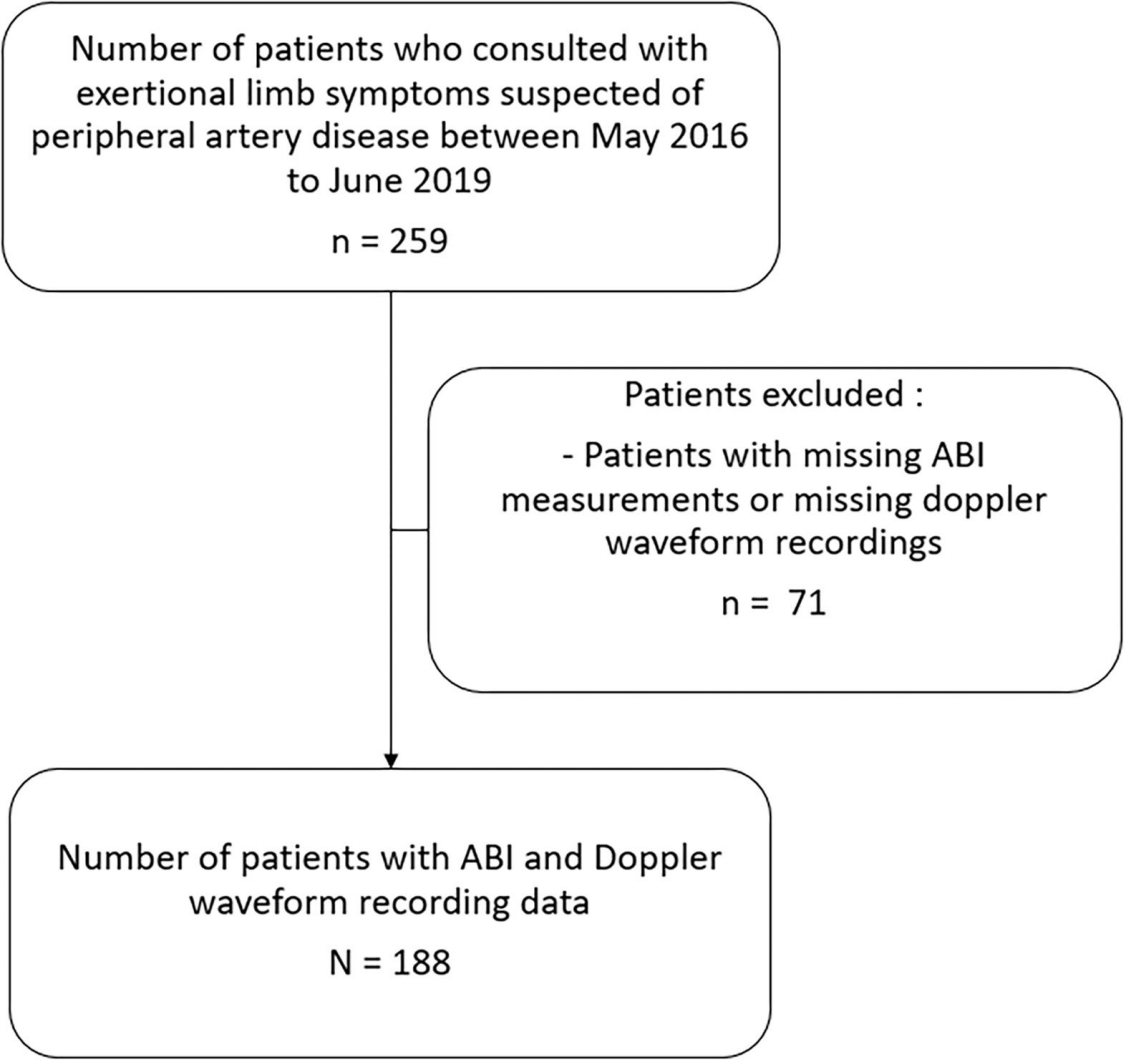
Diagram flow of the study. This figure shows the selection process of the study. Patients were excluded if there were missing ABI and missing arterial Doppler waveform. ABI means ankle brachial index.

Among 259 patients suspected of PAD with exertional limb symptoms, 188 patients were included as shown in Figure 1. Patients were excluded if there were missing limb artery pressures and Doppler waveforms for both arteries in each limb (*n* = 71).

Baseline characteristics are presented in Table 2. The average age was 62+/−12 years old and most of them were males (80.9%). The mean AHA-ABI was 0.88+/−0.30 for the right limbs and 0.88+/−0.26 for left limbs.

**Table 2:** Characteristics of the study population.

**Clinical characteristics**	***n*** **= 188**
Male, *n* = 188	152 (80.9%)
Age (years), *n* = 188	62 ± 12
BMI (kg/m^2^), *n* = 187	26.83 ± 4.52
Comorbidities, (history of), no. (%)	
Hypertension, *n* = 188	128 (68.1%)
Dyslipidemia, *n* = 188	127 (67.6%)
Diabetes mellitus, *n* = 188	38 (20.2%)
Tobacco, *n* = 182	
Active	74 (40.7%)
Stopped >6 months	80 (44.0%)
Never	28 (15.4%)
Vascular bypass, *n* = 184	27 (14.7%)
Vascular angioplasty, *n* = 182	56 (30.8%)
Myocardial infarction, *n* = 181	59 (32.6%)
Stroke (ischemic, hemorrhagic, or transient), *n* = 180	21 (11.7%)
Diuretics, *n* = 188	45 (23.9%)
ACEI/A2RA, *n* = 188	113 (60.1%)
Beta blockers, *n* = 188	62 (33.0%)
Calcium channel blockers, *n* = 188	48 (25.5%)
VKA, *n* = 188	12 (6.4%)
Oral anticoagulants, *n* = 188	8 (4.3%)
Antiplatelet agents (Aspirin or Clopidogrel), *n* = 188	147 (78.2%)
Statins, *n* = 188	118 (62.8%)
Fibrates, *n* = 188	4 (2.1%)

BMI, body mass index; ACEI, angiotensin-converting enzyme inhibitors; A2RA, angiotensin II receptor antagonists; VKA, vitamin K antagonists.

The Doppler waveforms were the same between ipsilateral limbs arteries in 138/188 in the right limb (73.4%) and 143/188 in the left limb (76.1%).

**Figure 2:**
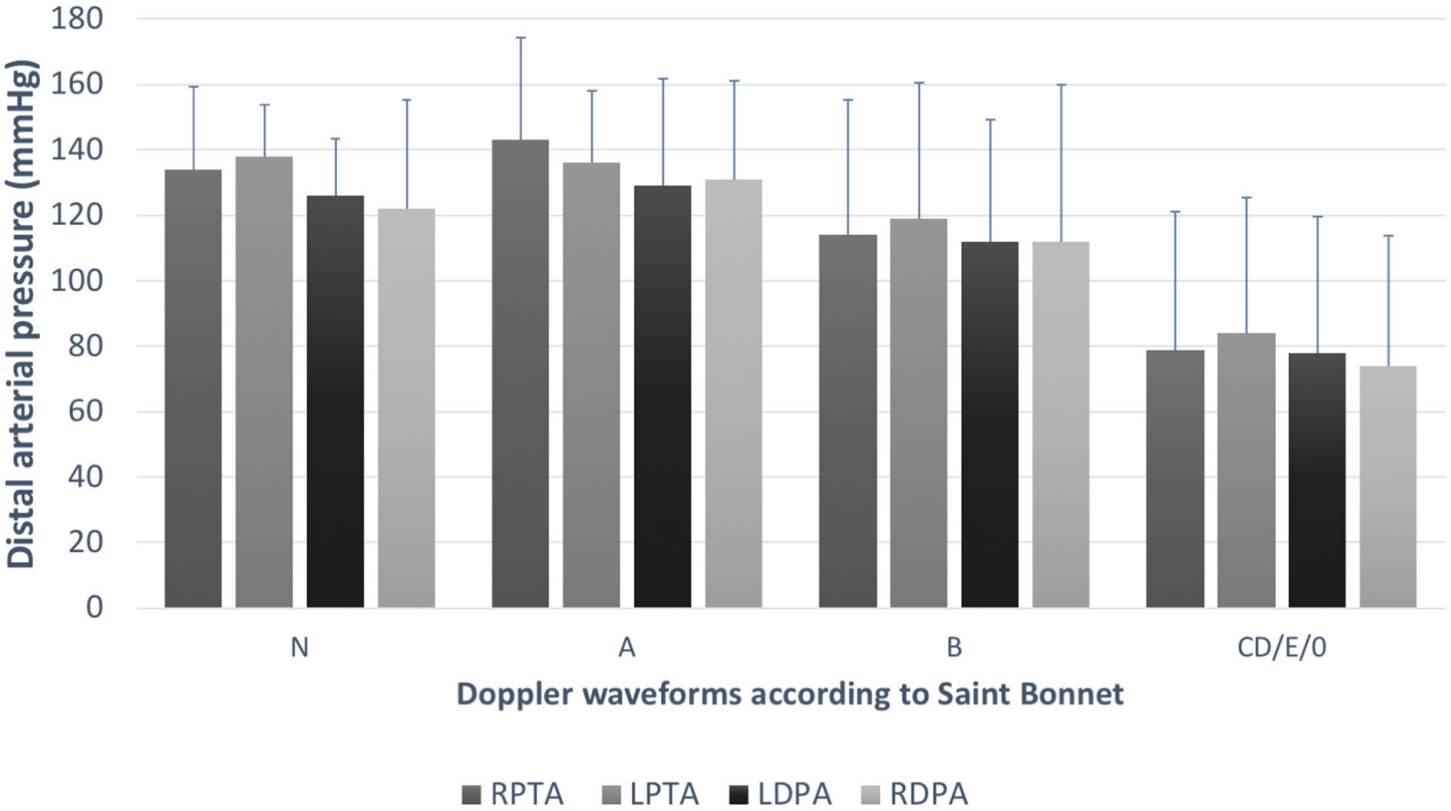
Distal pressures according to Doppler waveforms. This graph shows the mean arterial pressure (and the standard deviation) according to the Doppler waveform in each lower extremity artery from both legs. We can observe a trend of decreasing pressure with flow alteration. TPA, tibial posterior artery (RTPA,right TPA; LTPA, left TPA); DPA, dorsalis pedis artery (RDPA, right DPA; LDPA, left DPA).

Figure 2 shows the mean distal arterial pressure by artery on each leg according to the Doppler waveform (using the Saint-Bonnet classification).

Significant difference between the best Doppler waveform type (N according to the Saint-Bonnet classification) and the pressures from the arteries classed in the three last types of the classification (CD, E, and 0 among the Saint-Bonnet classification) was found with a *p*-value < 0.05 for each artery of each limb. However, no statistical difference appears between the arteries classified N and A (*p* > 0.05). When the *N* pressures are compared with the ones classified B, only the two tibial posterior arteries are significantly different.

The analysis of the concordance between the AHA-ABI and the FLOW-ABI shows an excellent concordance rating PAD/no PAD with a kappa value equal to 0.95 (95% CI: 0.90, 0.99) for the right limb and 0.91 (95% CI: 0.86, 0.97) for the left limb. The contingency tables are reported in Table 3. We obtain respectively for the right and left limbs only 5 (2.7%) and 8 (4.3%) discordant diagnosis between AHA-ABI and FLOW-ABI (please refer to Supplementary materials: Supplementary Tables 1, 2). When analyzing only the limbs with different Doppler waveforms, a perfect concordance was found for the right and left limbs. The kappa values were equal to 1.00 (95% CI: 1.00, 1.00) for the right limb and 1.00 (95% CI: 1.00, 1.00) for the left limb.

**Table 3:** Contingency tables between AHA-ABI and FLOW-ABI.

**Total limbs**							
**Right (*****n*** **= 188)**		**AHA-ABI**		**Left (*****n*** **= 188)**		**AHA-ABI**	
		**PAD**	No **PAD**			**PAD**	**No PAD**
FLOW-ABI	PAD	106	5	FLOW-ABI	PAD	103	8
	No PAD	0	77		No PAD	0	77
**Limbs with different types of Doppler waveforms according to Saint-Bonnet**							
**Right (*****n*** **= 50)**		**AHA-ABI**		**Left (*****n*** **= 45)**		**AHA-ABI**	
		**PAD**	**No PAD**			**PAD**	**No PAD**
FLOW-ABI	PAD	28	0	FLOW-ABI	PAD	29	0
	No PAD	0	22		No PAD	0	16

FLOW-ABI corresponds to the ABI calculated according to the best arterial flow. AHA-ABI corresponds to the ABI calculated according to the American Heart Association guidelines. PAD means lower extremity peripheral artery disease.

Medians of differences of ABI value between the two methods were 0.09 (IC 95%: 0.06, 0.16) for the right limb and 0.13 (IC 95%: 0.10, 0.16) for the left limb excluding one patient with outlier values. Indeed, one patient with diabetes had different values according to the two methods (0.45 vs. 1.57). The discordant patients' values are available in Supplemental materials.

**Figure 3:**
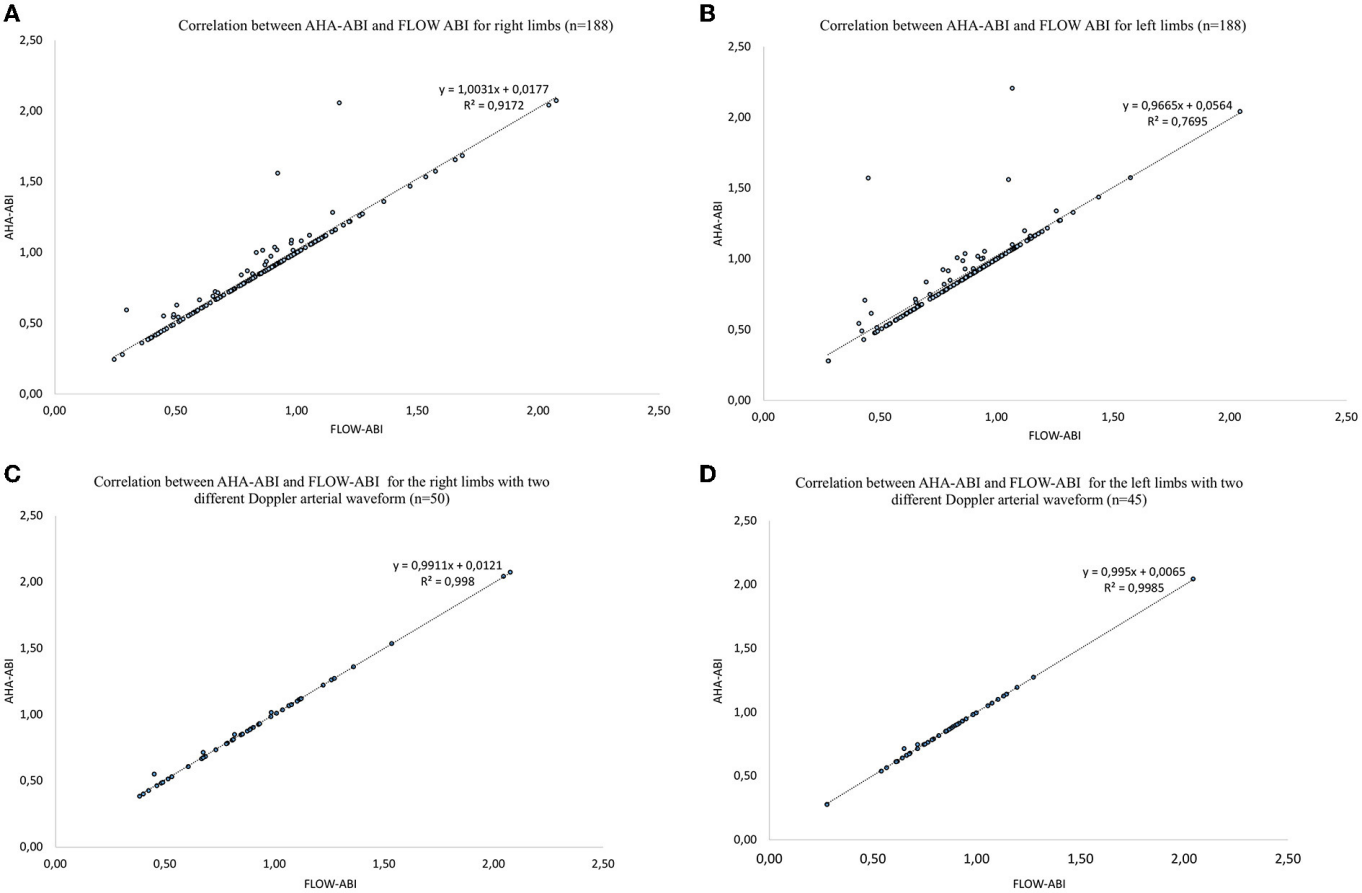
Correlation plots for each lower extremity between AHA-ABI and FLOW-ABI. PAD means lower extremity peripheral artery disease. FLOW-ABI corresponds to the ABI calculated according to the best arterial flow. AHA-ABI corresponds to the ABI calculated according to the American Heart Association guidelines. **(A)** Relationship between AHA-ABI and FLOW-ABI for the right limbs. **(B)** Relationship between AHA-ABI and FLOW-ABI for the left limbs. **(C)** Relationship between AHA-ABI and FLOW-ABI for the right limbs with two different Doppler arterial waveforms. **(D)** Relationship between AHA-ABI and FLOW-ABI for the left limbs with two different Doppler arterial waveforms.

We found a high correlation between AHA-ABI and FLOW-ABI on both lower extremities (Figure 3) with a Spearman's correlation coefficient of 0.96 for the right and 0.88 for the left *(p*-value < 0.0001). For the limbs with different waveforms, coefficients of correlation between AHA-ABI and FLOW-ABI were 0.99 and 0.99 for the right and leftlimbs, respectively.

## Discussion

Ankle-brachial index is an important diagnostic tool for PAD, but it is a time-consuming method with seven measures of pressure to perform when clinicians follow the AHA guidelines (23). To our knowledge, this study is the first that tries to simplify the ABI measurement to improve its use in clinical practice. We demonstrate an almost perfect concordance (kappa > 0.90) between AHA-ABI and FLOW-ABI for both lower extremities suggesting that both measurements can be used in clinical practice in patients suspected of PAD. Furthermore, it is important to note that discrepancies appear for only five right (2.7%) and eight left (4.3%) limbs. These discrepancies were in the usual variability (≤0.15) of the measure for 8 out of 13 (11, 28, 29). Of interest, the second analysis of limbs that had different Doppler waveforms types strengthens the results of the present result since the kappa value reached 1.00 for both limbs.

This result may have a strong impact in clinical practice by permitting the measurement of only one artery on each limb, the one with the best Doppler waveform, saving time and allowing the increased use of ABI, in particular among general practitioners. Indeed, physicians from various countries have not adopted, in common practice, this measurement in primary care practices (30, 31). The PAD Awareness, Risk, and Treatment New Resources for Survival (PARTNERS) program made evidence that physicians admitted the utility of ABI for screening the PAD but three main barriers were identified: the lack of time, staff availability, and reimbursement (12). The measurement time is a key point in the daily use of this test. The mean time for an ABI measurement was recently reported around 5 min by a trained vascular physician (32) without taking into account the 10-min rest time recommended (8). Another study found a mean time for ABI measurement by general practitioners around 17 min among more than 13,000 patients (33). Furthermore, this time constraint has an impact for vascular physicians who practice ABI several times a day. Considering a measurement time of 1 min per artery (34), measuring only five pressures instead of seven could save 2 min per patient, with similar accuracy. During a busy day, this could represent more than 30 min per day corresponding to more patients who can be seen.

The other interest of simplifying the ABI measurement might be about the education of medical students and residents. Indeed, studies have demonstrated that learning how to measure ABI is challenging (34) and not sustained (35). We hypothesize that this simplified method might improve the performance of medical students and residents to perform ABI, but this remains to be studied.

Moreover, this study confirms that the best pressure matches with the best Doppler waveform and conversely. This supports the importance of harmonization and definition of the arterial Doppler waveforms using a standardized classification. Indeed, various papers from different countries (USA, China, and France) found heterogeneity in Doppler waveform descriptions if no classification was used (7, 26, 36–38). This work was made possible by the use of the Saint-Bonnet classification that is recommended by the French Vascular Medicine Teachers (CEMV, College des Enseignants de Médecine Vasculaire) and the French Vascular Medicine and Surgery Societies (SFMV, SCVE) (7). Other classifications exist but a recent paper has shown that the Saint-Bonnet classification provides a superior categorization rate when compared to other classifications (24). Furthermore, the Saint-Bonnet classification was also associated with the functional status of patients with suspected PAD (31). In July 2020, the American Society for Vascular Medicine and Society for Vascular Ultrasound proposed a consensus (25, 26) about the description of arterial (and venous) Doppler waveforms to alleviate confusion. They developed this nomenclature based on the flow direction, the phasicity, and resistance. This description gathers the terms needed to use common language to be clinically useful. This consensus is of great interest, but studies about the use of the SVM/SVU consensus should be performed to confirm its utility in clinical practice.

### Limits

Our study has several limitations. First, the present results were found in patients with exertional limb symptoms and suspected of PAD. Thus, we cannot ascertain that similar results will be found in asymptomatic patients or in patients with critical limb ischemia. Second, the use of the posterior tibial artery by default in case of similar waveform categorization may be a bias. However, there is a trend in clinical practice to measure more pressure on the posterior tibial artery than on dorsalis pedis artery. Furthermore, although the artery caliber between both arteries does not appear significantly different in several studies (39, 40), there is a trend for an augmented prevalence of stenosis on the dorsalis pedis artery vs. the posterior tibial artery (40). Third, we followed the AHA procedure to measure the ABI except for the measurement of the brachial pressure that was measured with an automatic blood pressure monitor (Carescap Dinamap V100; GE Healthcare) as previously done in other papers (7, 22, 32). We are confident with our results since (i) Montgomery and Gardner (41) found no statistical difference between Doppler measurement and automatic measurement at the brachial level, and (ii) this is suggested by the French guidelines (7). Fourth, we did not study the reproducibility of the FLOW-ABI measurement, but this was not the aim of this study. However, the intra-observer coefficient in our team to perform AHA-ABI is 9.4% as previously reported (34). The reproducibility of the FLOW-ABI remains to be studied. Fifth, we had a small sample size of patients with diabetes, and data about chronic kidney disease (CKD) status was unknown in this population whereas ABI can be falsely elevated in diabetic and patients with CKD (42). Therefore, the results of this study should be used with cautious in these patients. Finally, this technique requires a machine that allows the visualization of the Doppler waveform. The price may be an issue in its development as well as the time required to train physicians on the machine's use and waveform interpretation and classification.

## Conclusion

Our study shows that ABI measurement using FLOW-ABI is as accurate as the AHA-ABI method for the diagnosis of PAD. The FLOW-ABI might replace the AHA-ABI in patients with exertional limb symptoms and suspected of PAD. Further studies with an external validation of these criteria seem useful to confirm our findings in a global population and the benefit in practice.

## Data availability statement

The raw data supporting the conclusions of this article will be made available by the authors, without undue reservation.

## Ethics statement

The studies involving human participants were reviewed and approved by Institutional Review Board from the University Hospital of Rennes (12, 17). The patients/participants provided their written informed consent to participate in this study.

## Author contributions

Study design: AM, GM, and ALF. Data collection: AM, QT, GM, and LO. Data analysis: AM, ELP, DL, and GM. Writing: AM and GM. All authors contributed to the article and approved the submitted version.

## Conflict of interest

The authors declare that the research was conducted in the absence of any commercial or financial relationships that could be construed as a potential conflict of interest.

## Publisher's note

All claims expressed in this article are solely those of the authors and do not necessarily represent those of their affiliated organizations, or those of the publisher, the editors and the reviewers. Any product that may be evaluated in this article, or claim that may be made by its manufacturer, is not guaranteed or endorsed by the publisher.

## Abbreviations

ABI: ankle-brachial pressure index at rest
PAD: lower extremity peripheral artery disease
TPA: tibial posterior artery (RTPA, right TPA; LTPA, left TPA)
DPA: dorsalis pedis artery (RDPA, right DPA; LDPA, left DPA).
