# Supplementary material for: Simplification of ankle-brachial-index measurement using Doppler-waveform classification in symptomatic patients suspected of lower extremity artery disease
Source: Front Cardiovasc Med. 2022 Sep 9;9:941600. doi: 10.3389/fcvm.2022.941600 (PMC9500167; doi:10.3389/fcvm.2022.941600)
Supplement: Supplementary file 1 [file Table_1.DOCX]

# **Supplemental materials**

**Table 1S. Discordant ABIs between AHA-ABI and FLOW-ABI**

| Right lower extremity (n=5) | | | | | Left lower extremity (n =8) | | | | |
| --- | --- | --- | --- | --- | --- | --- | --- | --- | --- |
| **FLOW-ABI** | | **AHA-ABI** | | **ABI difference** | **FLOW-ABI** | | **AHA-ABI** | | **ABI difference** |
| 0.87 | PAD | 0.91 | No PAD | 0.04 | 0.79 | PAD | 0.92 | No PAD | 0.13 |
| 0.88 | PAD | 0.94 | No PAD | 0.06 | 0.86 | PAD | 0.99 | No PAD | 0.13 |
| 0.83 | PAD | 1.00 | No PAD | 0.17 | 0.87 | PAD | 1.04 | No PAD | 0.17 |
| 0.89 | PAD | 0.98 | No PAD | 0.09 | 0.83 | PAD | 1.01 | No PAD | 0.18 |
| 0.86 | PAD | 1.02 | No PAD | 0.16 | 0.45 | PAD | 1.57 | No PAD | 1.12 |
|  |  |  |  |  | 0.90 | PAD | 0.93 | No PAD | 0.03 |
|  |  |  |  |  | 0.86 | PAD | 0.93 | No PAD | 0.07 |
|  |  |  |  |  | 0.77 | PAD | 0.92 | No PAD | 0.15 |

Table 1S: PAD means lower extremity peripheral artery disease. FLOW-ABI corresponds to the ABI calculated according to the best arterial flow. AHA-ABI corresponds to the ABI calculated according to the American Heart Association guidelines.

Table 2S. Characteristics of patients with discordant ABIs for each limb.

|  | **Right lower extremity (n = 5)** | | | | | **Left lower extremity (n = 8)** | | | | | | | | |
| --- | --- | --- | --- | --- | --- | --- | --- | --- | --- | --- | --- | --- | --- | --- |
| **FLOW ABI vs AHA ABI** | **0.87 vs 0.91** | **0.88 vs 0.94** | **0.83 vs 1.00** | **0.89 vs 0.98** | **0.86 vs 1.02** | **0.79 vs 0.92** | **0.86 vs 0.99** | **0.87 vs 1.04** | **0.83 vs 1.01** | **0.45 vs 1.57** | **0.90 vs 0.93** | **0.86 vs 0.93** | **0.77 vs 0.92** |  |
| ABI difference | 0.04 | 0.06 | 0.17 | 0.09 | 0.16 | 0.13 | 0.13 | 0.17 | 0.18 | 1.12 | 0.03 | 0.07 | 0.15 |  |
| Age (years) | 63 | 65 | 71 | 50 | 71 | 71 | 62 | 60 | 74 | 69 | 62 | 72 | 47 |  |
| Sexe (M/F) | M | M | M | M | M | M | M | M | M | M | M | M | M |  |
| Diabetes mellitus (yes/no) | no | no | no | yes | no | no | no | no | yes | yes | no | no | yes |  |
| Dyslipidemia (yes/no) | yes | no | yes | yes | yes | yes | yes | no | yes | yes | no | no | yes |  |
| Hypertension (yes/no) | yes | yes | yes | yes | yes | yes | yes | no | yes | yes | no | no | yes |  |
| Tabacco (active/stopped > 6 months/never) | active | active | stopped > 6 months | active | active | stopped > 6 months | stopped > 6 months | active | stopped > 6 months | stopped > 6 months | active | never | stopped > 6 months |  |

Table 2S: FLOW-ABI corresponds to the ABI calculated according to the best arterial flow. AHA-ABI corresponds to the ABI calculated according to the American Heart Association guidelines.
